# Moderate-Intensity Exercise Improves Body Composition and Improves Physiological Markers of Stress in HIV-Infected Men

**DOI:** 10.5402/2012/145127

**Published:** 2012-12-11

**Authors:** Wesley David Dudgeon, Jason Reed Jaggers, Kenneth Doyle Phillips, John Larry Durstine, Stephanie E. Burgess, George William Lyerly, John Mark Davis, Gregory Alan Hand

**Affiliations:** ^1^Department of Health, Exercise, and Sport Science, The Citadel, 171 Moultrie Street, Charleston, SC 29403, USA; ^2^Department of Exercise Science, Arnold School of Public Health, University of South Carolina, 1300 Wheat Street, Columbia, SC 29208, USA; ^3^College of Nursing, University of Tennessee, 4520 Ivy Rose Drive, Knoxville, TN 37918, USA; ^4^College of Nursing, University of South Carolina, 1601 Greene Street, Columbia, SC 29208, USA; ^5^Department of Health, Kinesiology, and Sports Studies, Coastal Carolina University, P.O. Box 261954, Conway, SC 29528-6054, USA

## Abstract

HIV/AIDS and its treatment often alter body composition and result in poorer physical functioning. The aim of this study was to determine the effects of a moderate-intensity exercise program on body composition and the hormones and cytokines associated with adverse health outcomes. HIV-infected males (*N* = 111) were randomized to an exercise group (EX) who completed 6 weeks of moderate-intensity exercise training, or to a nonintervention control group (CON). In pre- and postintervention, body composition was estimated via DXA, peak strength was assessed, and resting blood samples were obtained. There was a decrease in salivary cortisol at wake (*P* = 0.025) in the EX and a trend (*P* = 0.07) for a decrease 1 hour after waking. The EX had a significant increase in lean tissue mass (LTM) (*P* < 0.001) following the intervention. Those in the EX below median body fat (20%) increased LTM (*P* = 0.014) only, while those above 20% decreased fat mass (*P* = 0.02), total fat (*N* = 0.009), and trunk fat (*P* = 0.001), while also increasing LTM (*P* = 0.027). Peak strength increased between 14% and 28% on all exercises in the EX group. These data indicate that 6 weeks of moderate-intensity exercise training can decrease salivary cortisol levels, improve physical performance, and improve body composition in HIV-infected men.

## 1. Introduction

Over 33.3 million people are living with HIV-1 [1]. Since it was introduced in the mid to late 1990s, highly active antiretroviral therapy (HAART) has increased the time from HIV infection to acquired immunodeficiency syndrome (AIDS) diagnosis by 3 years and life expectancy of those with AIDS by up to 15 years [2]. In fact, these advances have allowed those living with HIV-1, and receiving treatment, to have life expectancies similar to uninfected persons with lifestyles that include smoking, heavy drinking, and obesity [3]. Mortality rates of HIV-infected persons dropped drastically within 18 months after the introduction of HAART, from 29.4 deaths per 100 person years to 8.8 deaths per 100 person years [4]. In fact, in younger (~30 years of age) persons with favorable disease markers (i.e., high CD4+ cell counts and low viral load) survival has been estimated at 31 years, with 45% of deaths attributable to non-HIV-related conditions, such as cardiovascular disease and cancer [5]. This brings about new challenges in treating persons with HIV-1, changing the focus from purely survival to improving quality of life by decreasing risk factors for other chronic conditions. Thus, it is important to understand how exercise training, which has been shown to be beneficial at reducing risk factors for both cardiovascular disease and cancer in uninfected populations, affects those persons living with HIV-1. 

While HAART has been able to significantly increase the survival of HIV-infected patients, HAART-associated cardiometabolic risks are increasing. HAART regimens, specifically those using protease inhibitors (PI), have been shown to cause lipodystrophy as well as metabolic changes that result in an increased risk for cardiovascular disease. It has been previously observed that patients with lipodystrophy show increases in total serum and low-density lipoprotein cholesterol, triglycerides, and insulin resistance [6]. 

A complication of HIV disease that has remained, even with the implementation of HAART, is HIV/AIDS-related wasting, which effects nearly 20% of those living with HIV-1 [7]. HIV/AIDS-associated wasting is characterized by reductions in fat mass, lean body mass (LBM), muscular strength, and functional performance and is associated with disease progression [8]. 

The pathophysiology behind these somatic changes increases the risk for cardiometabolic diseases possibly due to the proinflammatory process exhibited. Further, there is a strong association between HIV/AIDS-related wasting and increased mortality [9], impaired functional status [10], accelerated disease progression [11], and a declining ability to carry out activities of daily living [12]. HIV-infected persons also demonstrate an increased susceptibility to infection [13] and show a progressive depletion of metabolically active tissue near death [14]. 

The mechanisms behind HIV-associated wasting appear to be abnormal levels of circulating catabolic and anabolic hormones, cytokines, and binding proteins. Cortisol, which is elevated in HIV-infected persons [15–17], is a potent catabolic hormone, which increases rates of protein degradation and slows protein synthesis rates, especially in skeletal muscle [18]. Serum cortisol levels also appear to be the best predictor for insulin resistance followed by interleukin-6 (IL-6) in persons with inflammatory conditions [19]. Insulin-like growth factor 1 (IGF-1) is the mechanism by which growth hormone (GH) influences skeletal muscle and is a potent regulator of muscle mass [20]. Those persons suffering from HIV/AIDS wasting frequently demonstrate GH resistance, which manifests itself in increased GH levels and decreased IGF-1 levels, and may be attributed to malnutrition [21]. Insulin-like growth factor binding protein 3 (IGFBP-3) is the major IGF-1 binding protein in circulation and can extend the half-life of circulating IGF-1 from minutes to several hours thereby maintaining constant IGF-1 levels. HIV-1 infection increases the proteolysis of IGFBP-3, thus decreasing IGFBP-3 levels and in turn reducing the amount of IGF-1 in circulation [22]. Another hormone of interest is testosterone, which is low in 30–50% of HIV-infected men [23] and is of significance because testosterone is a potent regulator of skeletal muscle mass that acts, via multiple pathways, to increase protein synthesis [24–26]. 

Inflammation is a prominent component in HIV/AIDS wasting and is also linked to cardiometabolic conditions. While inflammation is a necessary process for muscle generation [27], it may be catabolic if found in excess levels for prolonged periods. Cytokines of note in this inflammatory process include tumor necrosis factor alpha (TNF-*α*), interleukin one beta (IL-1-*β*), and IL-6. However, IL-6 may also exhibit anti-inflammatory functions, as it can inhibit TNF-*α* and IL-1-*β* [28]. TNF-*α* has been associated with muscle wasting in many diseased populations [29–31] including HIV-1 [32] and HIV-infected persons have been shown to have elevated TNF-*α* levels [33, 34].

Elevated levels of these proinflammatory markers are also known to be associated with an increased risk of chronic disease. Relationships have consistently been identified among atherosclerosis and cardiovascular disease with greater levels of C-reactive protein (CRP), TNF-*α*, and IL-6 [35, 36]. Further, IL-1-*β* has been implicated in numerous disease states including rheumatoid arthritis [37], atherosclerosis [38], and type I diabetes [39]. Studies looking at patients with metabolic disorders have also shown increased levels of IL-6 and CRP among type II diabetics, obese individuals, and patients experiencing metabolic syndrome [40–42]. 

An emerging treatment that has the potential to improve the circulating abnormalities (e.g., low anabolic hormone levels and high proinflammatory markers) of those with HIV-1 is exercise training. Before further discussing the potential beneficial effects of exercise in HIV-infected populations, it should first be recognized that research has shown exercise to be safe in this population. Low- to moderate-intensity exercise does not alter CD4 cell counts or viral load, nor does it increase the prevalence of opportunistic infection [43]. Additionally, no reports of adverse side effects to exercise interventions have been reported.

Resistance exercise training is known to increase skeletal muscle mass and muscular strength in numerous healthy and clinical populations regardless of gender, age, or race [44]. Aerobic exercise is also known to prevent or manage chronic diseases such as insulin resistance, cardiovascular disease, and cancer. This could possibly be a result of the protective effects aerobic exercise has shown to have against inflammation. Studies have repeatedly shown the benefits of aerobic exercise against inflammation by decreasing CRP, IL-6, TNF-*α*, among other proinflammatory markers [45–47]. Further, limited data in HIV-infected populations have shown similar effects. Progressive resistance exercise alone has produced significant increases in muscular strength and lean body mass in HIV-infected persons with and without wasting [48–50]. Similarly, the combination of resistance exercise and aerobic exercise has also increased strength and lean mass in HIV-infected populations [51–53] and produced transient increases in anabolic hormones [54]. Thus, it appears that resistance exercise training has the potential to be a complimentary therapy to pharmacological treatments for maintaining and increasing lean body mass and muscular strength in HIV-infected persons.

However, what remains to be seen is the effect of a lower dose (lower volume and lesser intensity) of combined resistance and aerobic exercise on body composition, strength and inflammation in HIV-infected persons. Further, this type of intervention may improve subject compliance. Therefore, the aim of this study was to determine the effect of an American College of Sports Medicine- (ACSM-) based low-volume, moderate-intensity resistance and aerobic exercise training program on the strength, body composition, and circulating hormone and inflammatory cytokine profile in a sample of HIV-infected men.

## 2. Methods

### 2.1. Sample

HIV-infected men over 18 years of age free of any known opportunistic infections, currently receiving antiretroviral therapy (ART), and physically able to complete the exercise intervention were recruited from local HIV/AIDS service organizations in the Columbia, SC metropolitan area. Flyers were placed in the waiting areas of clinics, and research team members enlisted the assistance of HIV/AIDS case workers in recruiting subjects. 

Individuals who met the above criteria were excluded from the study following a physician's screening if (1) their medical history revealed current opportunistic infection(s), (2) they were found to be currently using, or had used in the past, hormone therapy, (3) they scored 5 or greater on the Drug Abuse Screening Test (DAST) and/or the Michigan Alcohol Screening Test (MAST), (4) they were found to be currently involved in a structured exercise program, or (5) any contraindications for graded exercise stress testing, as specified by the American College of Sports Medicine [55] were identified. 

### 2.2. Procedure

All procedures, including testing and exercise training, were completed in the Clinical Research Laboratory in the Department of Exercise Science at the University of South Carolina. After signing the informed consent statement, subjects were assigned an identification number and then were assisted in completing a demographic data sheet (age, race, CDC disease stage, and current health status) and a cardiovascular risk factor survey that addressed current medications (including HIV medications), family history of cardiovascular disease, smoking habits, physical activity habits, any significant health concerns (other than HIV infection), and other pertinent health information. Medical personnel then reviewed the risk factor survey, and if no contraindications for exercise stress testing were identified, subjects were cleared to receive a graded treadmill stress test (GXT). Following successful completion of the GXT, subjects were randomized, using a random number table, to the intervention group (EX) or a nonintervention control (CON) group. Following the completion of study requirements, those randomized to the CON group (who did not receive intervention) then received the aerobic and resistance exercise intervention twice weekly for six weeks. The Office of Research Compliance at the University of South Carolina approved the study and its procedures.

### 2.3. Body Composition Assessment

Dual energy X-ray absorptiometry (DXA) scans (GE Medical Instruments, Madison, WI) for body composition were administered before the first exercise session and within 1-week following the last exercise session. A state certified DXA technician calibrated the equipment, performed each scan, and analyzed the results yielding lean mass and fat mass data, both for the entire body and by region. 

### 2.4. Peak Strength Assessment

All subjects had peak strength determined on upper body resistance exercises (chest press, lat pull down) and lower body resistance exercises (leg extension, leg curl). Subjects were instructed on the proper techniques for each exercise and then were given one warm-up set of 8–12 repetitions (minimal weight was selected for this set) to become comfortable with the exercise. Subjects were then instructed to complete three repetitions on each exercise with the most weight possible while maintaining proper form. Weight selection was based on subject input and observation by the investigator. Since this population was untrained and unfamiliar with resistance training, a three-repetition maximum (3-RM) was used instead of the standard 1-RM, which has been used by others in clinical populations [7, 56, 57]. Using the standard conversion set forth by Brzycki [58], a 3-RM is approximately 94% of the 1-RM. Sixty percent of this 3-RM was then used as the initial weight for the resistance exercises.

### 2.5. Design

A randomized control group experimental design was used to determine the effects of moderate-intensity exercise training on circulating hormone, cytokine, and binding protein levels in HIV-infected men. All EX subjects were scheduled to attend 2 exercise sessions per week during the 6-week study. Exercise sessions were separated by at least 48 hours to allow for adequate recovery. Subjects had to complete 10 of the 12 sessions over the study duration to be included in the final analysis. Following the last exercise or control session, subjects were given three salivettes to take home. They were instructed to collect saliva samples immediately after waking (W), 1 hour after waking (W+1), and 2 hours after waking (W+2) on the day they were scheduled to return for posttesting. On the pre- and posttesting days, subjects were instructed to arrive fasted and were scheduled to arrive at the same time of the day so as to avoid hormonal variation due to normal cycles. Blood was collected and saliva samples were returned at this time.

### 2.6. Exercise Intervention

Those subjects randomized to the EX group completed 30 minutes of aerobic exercise training, on a treadmill or stationary cycle, in the intensity range of 60–75% of their age predicted maximum heart rate (208 × 0.7 (age in years)) twice weekly, for 6 weeks. Heart rate during exercise was monitored using Polar Heart Rate Monitors. Each exercise session consisted of a 3–5 minute warm up period followed by 30 minutes of training within the prescribed intensity range, and then a 3 to 5 minute cool down. Treadmill speed and grade were adjusted during each session to keep subjects within their prescribed intensity range.

Following aerobic exercise subjects completed upper body and lower body resistance training exercises. Movements targeting the chest, upper back, triceps, upper anterior and posterior legs, and lower legs were performed on resistance training machines, while movements targeting the biceps brachii and deltoids were performed using free weights. Sets were alternated in a circuit such that one particular muscle group was not exercised in consecutive sets. Subjects were given approximately one minute recovery time between sets. Resistance was adjusted so subjects could complete 12 repetitions per set of each exercise while maintaining proper form. As strength increased, resistance was changed so as to keep the subjects at the prescribed training intensity. Exercise training sessions were separated by at least 48 hours to allow for recovery and a trained exercise physiologist monitored all exercise sessions. 

### 2.7. Sample Analysis

#### 2.7.1. Saliva

Saliva was collected using salivettes, which contain small cotton swabs. Subjects were given the cotton swabs and instructed on appropriate placement of the swab in their mouths. The saliva was recovered by inserting a saturated cotton swab into the salivette and centrifuging the container at a relative centrifuge force of 39,000 kg for 5 minutes. The particulate free saliva sample was then obtained from the bottom of the salivette and saliva aliquots were frozen and stored at −80°C until analyzed. Salivary cortisol (CORT) was analyzed using a colorimetric sandwich ELISA (R&D Systems, Minneapolis, MN, USA). 

#### 2.7.2. Blood

Blood (3 mL) was collected at the specified times into serum separator tubes via venipuncture in an antecubital vein in the lower arm. All blood samples were taken with subjects in a seated position. The remaining whole blood was then centrifuged at a relative centrifuge force of 39,000 kg for 20 minutes, and the resulting supernatant was removed, alliquoted, and frozen at −80°C until needed for analysis. Commercially available colorimetric sandwich enzyme immunoassays (R&D Systems, Minneapolis, MN, USA) were used for the analysis of IL-6, sTNFrII, IGF-1, IGFBP-3, and GH. IL-1*β* was measured with a solid phase enzyme amplified sensitivity immunoassay (BioSource Europe, Nivelles, Belgium).

#### 2.7.3. Urine

Urine was collected in urine collection containers over 24-hour periods in which no exercise was performed, both before and after the 6-week exercise training period. Subjects were given the urine collection container and instructed on the proper procedures for collection. After waking on the day in which the urine was to be collected, the first urination of the day was not collected, but each subsequent urination for the next 24-hours was to be collected in the urine collection container. Subjects were instructed to keep the urine cool until returned to the research facility, at which time it was alliquoted and frozen at −80°C until needed for analysis. 

GH and CORT are released in a pulsatile fashion into the circulation throughout the day [59]. Therefore, to better estimate the total amount of hormone released, these hormones were measured in one milliliter samples of the urine, which provides the amount per milliliter. This value was then multiplied by the volume of urine produced during the day to yield the total amount produced over a 24-hour period. Urinary CORT and GH were both analyzed using a colorimetric sandwich ELISA (R&D Systems, Minneapolis, MN, USA). Normal urine production varies greatly between individuals, and is influenced by many factors. For the purposes of this study, only those subjects who collected at least 500 mL of urine (1/3 the mean production for participants) at each time point were included in the final analysis. 

### 2.8. Quantification of Exercise Protocol

Caloric expenditure of physical work was quantified during the aerobic exercise portion of the exercise protocol using the formula published by Keytel et al. [60]. This method estimates caloric expenditure per minute of activity based on the heart rate recorded during the previous minute and includes the age, gender, body weight, and VO_2_ max of the subject. The calories expended per minute were then multiplied by the duration of the exercise session yielding the total calories burned per session. There is no accepted method of estimating the caloric expenditure of resistance exercise, therefore the resistance portion of the exercise protocol was quantified based on total weight moved per session.

### 2.9. Measurement of Physical Activity

The Physical Activity Scale for the Elderly (PASE) was used to assess physical activity levels to determine if the aerobic and resistance exercise program had an effect on leisure and occupational physical activity levels in the exercise group. The PASE, which asks the participant to recall their activities over the previous 7 days, was administered at the pretesting session and again at the posttesting session. 

### 2.10. Statistical Analysis

Repeated measures analysis of variance (ANOVA) was used to compare salivary cortisol within groups. Tukey post hoc analysis of treatment means was used to identify differences between groups. All other within-group variables were assessed using a paired *t*-test and comparison of variables between groups was done with an unpaired *t*-test. The significance level of all statistical tests was set at *α* = .05. All values are expressed as mean ± standard error (SE). Upon completion of the initial analysis, the exercise group data was divided at the median body fat percentage, which was 20% body fat, and the data from these two groups was analyzed. 

## 3. Results

### 3.1. Demographic Data

One hundred eleven (111) HIV-infected males were cleared for study participation while 17 subjects were excluded at screening based on the previously mentioned criteria. After oversampling of the control group and subject attrition, 59 subjects were randomly assigned to the control group and 52 subjects were randomized to the EX group. Forty (40) subjects completed the exercise intervention and 36 subjects in the control group finished the study. Body composition data were collected on 31 subjects in the exercise group and 27 persons in the control group. Hormone data were collected on 16 subjects in the exercise group and 27 persons in the control group. Demographic data for each group are presented in Tables 1 and 2. 

### 3.2. Quantification of Exercise Protocol

The number of calories burned per week of aerobic exercise was not different between weeks; it ranged from a high of 523.3 ± 41.1 calories during week 4 to a low of 467.3 ± 54.5 calories during week 6. There was a difference in the total weight moved during each week of the resistance exercise portion of the protocol, with total pounds increasing over time. As expected, the total pounds lifted during week 6 was greater than week 1 (*P* < .001), week 2 (*P* < .001), week 3 (*P* < .001), and week 4 (*P* = .02). Similarly, the pounds lifted during week 5 were greater than week 1 (*P* < .001), week 2 (*P* < .001), and week 3 (*P* = .005). 

### 3.3. Physical Activity Measurement

Following the 6-week moderate-intensity exercise intervention, there was no change in leisure and occupational physical activity levels (as measured by the PASE) in either the exercise group (*P* = .38) or the control group (*P* = .42). Additionally, there were no differences in baseline leisure and occupational physical activity levels between the exercise and control groups (*P* = .29).

### 3.4. Hormone and Cytokine Variables

**Figure 1:**
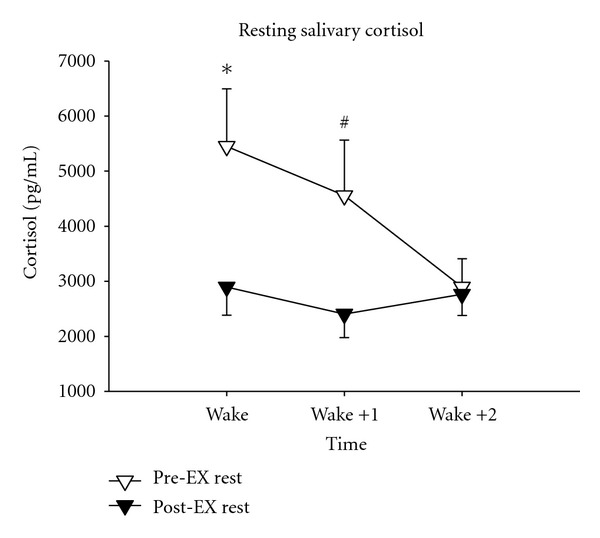
Salivary cortisol in exercise group. Salivary cortisol in MOD group (*N* = 13) subjects measured immediately after waking, 1 hour after waking, and 2 hours after waking. *Indicates difference from Pre-EX test with *P* < .05.  ^#^Indicates difference from Pre-EX test with *P* = .07.

There was a decrease in salivary cortisol at wake in the exercise group following the intervention, and there was a trend for a decrease one hour after waking in the exercise group (see Figure 1). There were no other changes from pre- to postintervention in either the exercise group or the control group in any of the hormone and cytokine variables measured (see Table 3).

### 3.5. Body Composition

**Figure 2:**
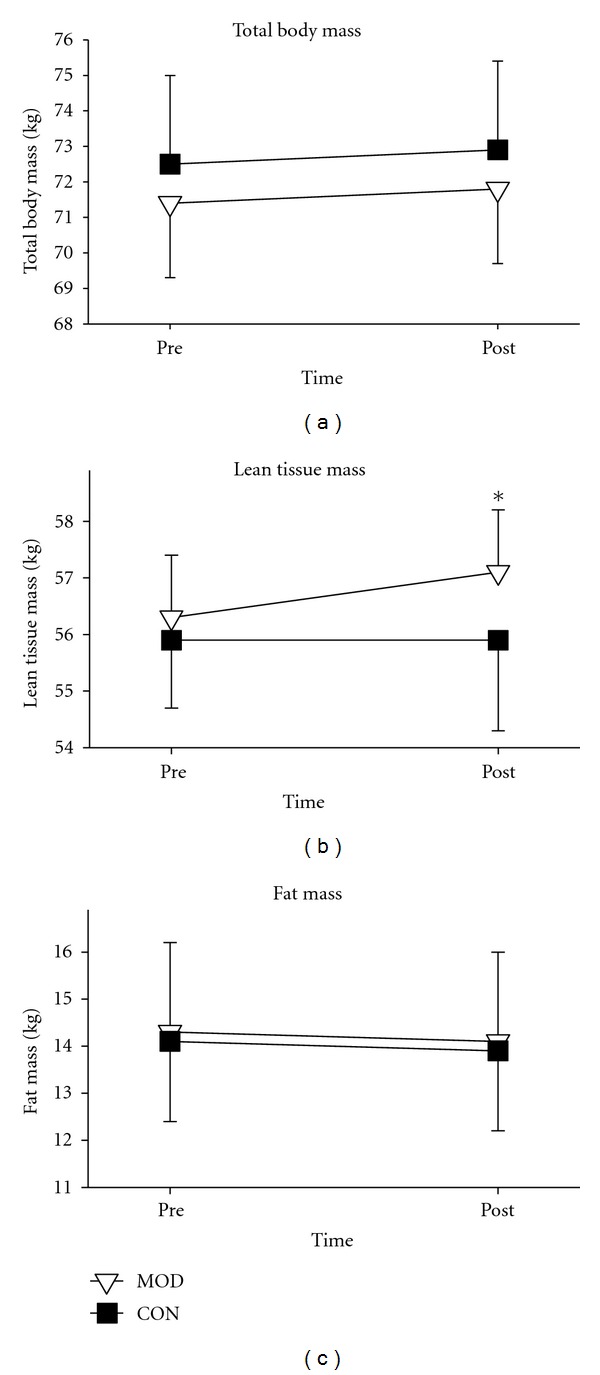
Lean tissue, fat, and total body mass of exercise and control group subjects. Body composition measurements taken via DXA in the exercise (*N* = 31) and control (*N* = 27) group before (pre) and after (post) the 6-week exercise intervention. *Indicates a significant change in the exercise group from Pre- to post-test.

At study initiation, there were no differences between groups regarding any of the body composition variables, including body mass index (see Table 4). Following the training period, the exercise group had an increase in lean tissue mass, while no change was seen in the control group (see Figure 2). Neither group exhibited changes in fat mass, total percentage of body fat, trunk percentage of fat, or any other body composition variable measured.

**Figure 3:**
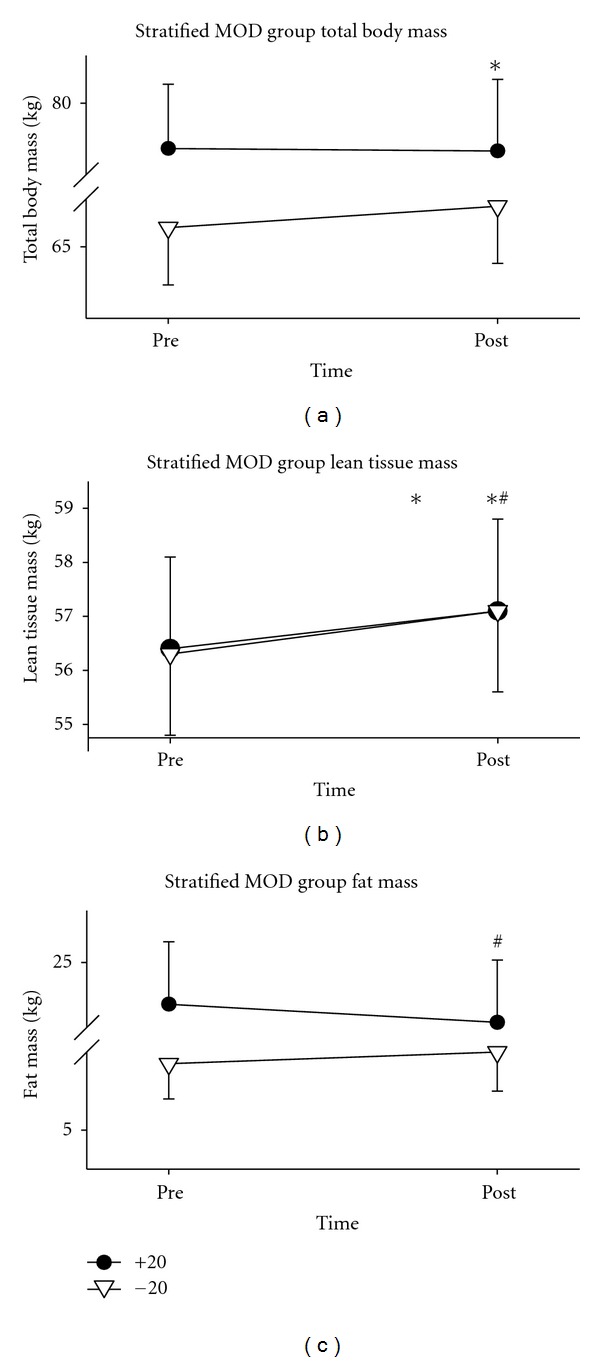
Lean tissue, fat, and total body mass of exercise subjects above and below 20% body fat. Body composition measurements taken via DXA in the +20 (*N* = 14) and −20 (*N* = 17) exercise groups before (pre) and after (post) the 6-week exercise intervention. *Indicates a significant change in the −20 group; ^#^indicates a significant change in the +20 group.

**Figure 4:**
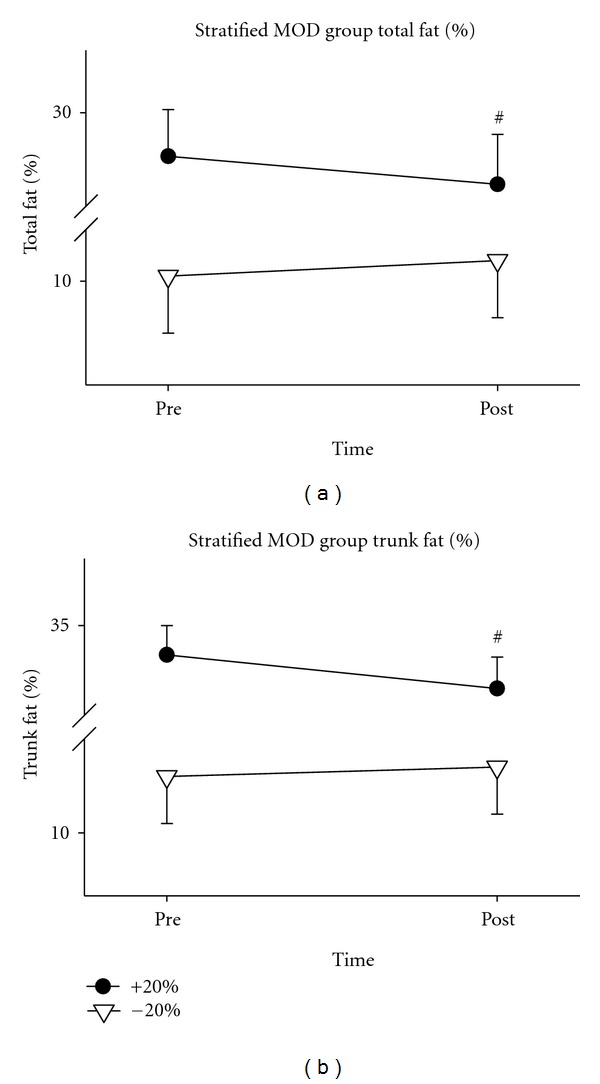
Total percentage of body fat and trunk percentage of body fat in exercise subjects above and below 20% Body Fat. Body fat percentage of measurements taken via DXA in the +20 (*N* = 14) and −20 (*N* = 14) exercise groups before (pre) and after (post) the 6-week exercise intervention. ^#^Indicates a change in the +20 group.

The exercise group was then divided at the median body fat percentage: those with a preintervention body fat above 20% (+20), and those below 20% (−20) (see Table 5). Following the intervention, subjects in the +20 group exhibited a significant increase in lean tissue mass (*P* = .027), as did the −20 group (*P* = .014). Further, the +20 group decreased total fat mass (*P* = .02), percentage of total body fat (*P* = .009), and percentage of trunk fat (*P* = .001). No changes in these variables were seen in the −20 group. However, the −20 group did show an increase in total body mass (*P* = .007). The −20 group also had a significant increase in BMI (*P* = .007) following the intervention, while no change was observed in the +20 group (see Figures 3 and 4).

### 3.6. Peak Strength

Following the intervention, both the exercise and the control groups showed an increase in lower body strength. The exercise group had an increase in peak leg extension strength (*P* < .001) and an increase in peak leg curl strength (*P* = .001), while the control group also had increase in leg extension (*P* = .03) and leg curl strength (*P* = .006). The control group, however, did not show a change in lat pull down strength or chest press strength, while the exercise group did improve both lat pull down (*P* < .001) and chest press strength (*P* < .001) following the intervention (see Table 6).

### 3.7. Girth

There were no detectable changes in the exercise group for arm girth, leg girth, or chest girth following the training session. Additionally, there were no changes in arm girth, leg girth, or chest girth in the control group. Further, there were no differences between the two groups in any of the three girth measurements. 

## 4. Discussion

Recent work has suggested that aerobic and resistance exercise interventions can cause beneficial physiologic responses and adaptations in HIV-infected populations [48, 61–63]. Specifically, we have shown that ACSM-based moderate-intensity aerobic and resistance exercise can cause transient increases in anabolic factors (GH) and decreases in catabolic factors (sTNFrII). Thus, the goal of this study was to determine if 6-weeks of a similar ACSM-based exercise program caused body composition, hormonal, and performance benefits. The rationale for testing this intervention is that (1) it is of shorter duration than other published works, (2) it is of lower intensity than other published works, (3) it combines both aerobic and resistance exercise, (4) it is an industry-recognized exercise prescription that benefits cardiovascular health in the apparently uninfected population, and (5) it has already been shown to stimulate positive humoral changes in an HIV-infected population [61]. 

There have been few original randomized controlled studies that have examined the effects of progressive resistance exercise interventions in HIV+ populations [48, 49, 64, 65]. Fewer yet are studies combining progressive resistance training and aerobic training [51–53]. However, results have been encouraging, with increases in total body mass [48, 51, 52, 65], lean mass [48, 49, 51], and peak strength [48, 49, 51–53, 65] being reported.

The majority of these interventions have required subjects to participate in three exercise sessions per week, and with the exception of one study [65], all have been 8–16 weeks in duration, with 12 weeks being the most common [48, 51–53]. The interventions have generally required the subjects to start resistance exercise at 60% of their 1-RM, then increased resistance to anywhere from 80% to 90% 1-RM near the end of the study. 

### 4.1. Body Composition

The effect of exercise training on total body mass in HIV-infected persons has been ambiguous, with some showing an increase [48, 52, 65], while others [49, 51] have failed to detect a change. We did not show a difference in total body mass following training. Results similar to our findings were reported by Grinspoon and colleagues [51] and Roubenoff and colleagues who did not show an exercise effect on total body mass following 8 weeks of exercise training at intensities of 80% 1-RM and higher. Conversely, Spence et al. [65] reported a 1.7 kg increase in body weight following 6 weeks of three weekly progressive resistance exercise sessions. However, these subjects had recently recovered from *Pneumocystis carinii* pneumonia and were examined before HAART was available. Similarly, Lox and colleagues [52] reported a 2.2 kg increase in total body mass following 12 weeks of three weekly progressive resistance exercise sessions in men receiving ART. A 2.2 kg increase in total body mass was also seen by Bhasin et al. [48] in men (all receiving ART) following 16 weeks (3 sessions/wk.) of progressive resistance exercise with resistance progressing from 60% 1-RM to 90%. The discrepancy in total body mass changes following exercise is difficult to interpret for the following reasons: subjects in all the above studies had similar total body mass at study initiation, study duration did not appear to have an effect as both shorter [65] and longer [48, 52] durations showed increases, lower progressive resistance exercise intensities [65] and higher intensities [48] produced changes, and changes were seen in those who were on ART [48] and who were not [52, 65]. 

Our detection of a 0.8 kg increase in lean tissue mass is consistent with the findings of others. Grinspoon and colleagues [51] (2.3 kg), Bhasin and colleagues [48] (2.0 kg), and Roubenoff and colleagues [49] (1.8 kg) all showed significant increases in lean tissue mass using DXA, while Lox [52] showed a 2.3 kg increase in nonfat tissue using the skin-fold technique. While our change is significant, it appears to be less than the other studies mentioned. However, it should be noted that the subjects in our study trained at a lower intensity, less frequently, and for a shorter time period than the other studies showing increases in lean tissue mass. Of the other three studies mentioned, only Roubenoff et al. [49] (8 weeks) trained subjects for less than 12 weeks, and the reported change in lean tissue mass (1.8 kg) was less than the other studies (2.3 kg, 2.3 kg, and 2.0 kg) of longer duration. Additionally, of the other studies mentioned, only Lox et al. trained subjects at a lower intensity (60% 1-RM), but still observed similar lean tissue mass gains. It appears that ART does not influence lean tissue mass gains, as those subjects not using ART [52] and those subjects using ART [48, 49, 51] both showed an increase in lean tissue mass. However, it appears that the length of the intervention may affect lean tissue mass gains, in that interventions of longer duration may result in larger lean tissue mass gains. 

To our knowledge, we are the first to stratify subjects based on preintervention body fat percentage. The rationale for this action was based on our finding of a significant increase in lean tissue mass in the exercise group, with no changes in total body mass or fat mass. When looking at the exercise group in this fashion it can be seen that the +20 did not change total body mass and the −20 gained total body mass, thus explaining the no change in total body mass the entire exercise group. Similarly, the +20 group lost total fat mass while the −20 group did not change in these variables, so again no overall change was seen. This suggests a differential effect of exercise training based on preintervention body composition.

The only group to show a regional change in LTM was the −20 group, who exhibited an increase in leg lean tissue mass. Using CT, Grinspoon et al. [51] showed an increase in thigh muscle area, and using MRI, Bhasin et al. [48] found and increase in thigh muscle volume. Though no direct mass measurements were taken, Spence et al. [65] showed an increase in thigh girth. Thus, it seems consistent across the literature that resistance training is capable of increasing lean tissue mass in the legs. However, what remains uncertain is why we only found an increase in leg lean tissue mass in the −20 group, and not the +20 group. 

Only Roubenoff et al. [49] reported a decrease in total fat mass following their exercise intervention. This change in fat, however, could stem from the extended exercise time prescribed in the study (20 minutes of aerobic training in addition to 1 hour of resistance training). Our study, along with the remainder of the studies included in this discussion, only prescribed exercise regimens lasting approximately 60 minutes. The 0.92 ± 2.22 kg decrease in total fat mass reported by Roubenoff et al. [49] is similar to the 0.73 ± 0.3 kg decrease seen in our study. However, the decrease in our study was only observed in men with body fat above 20%. A possible explanation for this could be that the subjects in the Roubenoff et al. [49] study had 20.3 ± 2.3 kg of body fat at study initiation, while our exercise group as a whole had only 14.3 ± 1.9 kg, and our +20 group had 23.4 ± 2.4 kg. Therefore, when similar populations are compared, it appears that the exercise effect on total body fat is consistent. Further, our finding of a decrease in trunk fat in the +20 group suggests additional health benefits, as trunk fat is associated with numerous cardiometabolic diseases which are common in HIV disease [5].

### 4.2. Physical Functioning

Following our exercise training program, those in the exercise group showed significant increases in all four strength exercises tested: leg extension (25 ± 6.2%), leg curl (27.6 ± 9.5%), lat pull (13.8 ± 3.3%), and chest press (14.3 ± 1.8%). Bhasin et al. [48] (29–36%), Roubenoff et al. [49] (31–50%), and Lox et al. [52] also showed increased strength using conventional isotonic testing equipment. Rigsby et al. [53] showed upper and lower body strength increases using a hydraulic dynamometer. Only Grinspoon et al. [51] failed to show a change in strength following the exercise intervention; however, this study used isometric testing which, as the authors suggest, may not have detected a strength change if it existed [66]. These findings indicate that, even at moderate intensities, resistance exercise training can cause an increase in strength in HIV-infected men, independent of ART usage. 

### 4.3. Resting Hormones and Cytokines

The only change detected within groups at the conclusion of the intervention was a decrease in salivary cortisol from pre- to posttest in the exercise group at the waking time point, and there was a trend for a decrease one hour after waking. There were no other changes detected in CORT, TEST, IGF-1, IGFBP-3, GH, IL-6, STNFrII, or IL-1-*β* in the CON or exercise groups. 

In light of the changing landscape of HIV disease, namely, the shift towards a chronic disease, it is important to determine how HIV-infected persons who have yet to develop AIDS respond to exercise training. This population is now dealing with the same chronic diseases as their noninfected counterparts, specifically cancer and cardiometabolic diseases which are the leading non-HIV related causes of death [5]. While certain causes of these diseases (physical inactivity, stress, poor diet, etc.) undoubtedly are involved in their development in HIV-infected persons, the chronic immune activation and inflammatory state found in these persons may also be contributing factors [67]. Thus, it is prudent to examine exercise training in this population given the effect of exercise on body composition, hormone, and cytokine profiles, as well as the established benefits of exercise in reducing the risk for developing cardiometabolic diseases and cancer.

 There is no research outlining the effects of exercise training on resting hormone and cytokine levels in HIV-infected persons. However, data does exist in other populations suggesting that exercise can influence many of the anabolic and catabolic factors implicated in the altered metabolic environment of those with HIV [68, 69]. The measurement of resting cytokines in the circulation is difficult, thus little research in this area is available. One of the major factors influencing cytokine levels is immune function, thus, whether the individual is currently ill or recovering from illness, cytokine levels are not “normal” [70]. Further, it appears that the primary impact of exercise on cytokine levels occurs during, and immediately following exercise sessions.

IL-6 levels have been shown to increase immediately following exercise that is not of sufficient intensity to induce muscle damage [71, 72] and this increase may provide anti-inflammatory effects [28]. This is the proposed rationale for the association that has been discovered between increased physical activity levels and decreased systemic inflammation in healthy persons [73–76]. Though we did see elevated resting levels of one inflammatory marker (IL-1-*β*) in our population, we did not see a change in IL-6 following exercise. It should be noted that IL-6 levels (3.6 ± 0.7 pg/mL) were found to be within a normal range in our sample and we have previously shown that IL-6 levels rise acutely following exercise [54], thus fitting with the hypothesis that the anti-inflammatory effects of exercise in this population are the result of acute changes. 

We anticipated a change in sTNFrII and IL-1-*β* following the exercise intervention given that basal levels in HIV-infected populations have been shown to be elevated and acute exercise appears to decrease these levels [77]. We found sTNFrII to be within normal range in our population, thereby potentially explaining our lack of a detectable change because these levels were not elevated. By contrast, we found resting IL-1-*β* to be well above normal, which would fit with other literature. However, the theoretical anti-inflammatory effects of exercise are dependent upon another cytokine, IL-6, which we also found not to change following our training. Thus, if IL-6 did not increase, it would not directly block TNF-*α* and IL-1-*β* production, nor would it inhibit these inflammatory cytokines by inducing production of the anti-inflammatory agents IL-10 and IL-1ra production.

Normal daily urine production is highly variable and can range 700–2000 mL/day, with males typically producing approximately 1500 mL/day. The subjects in our study averaged 772 ± 118 mL/day, with a range from 300 mL to 2800 mL. We had access to our subjects only during the testing days, and the exercise sessions, thus in order to get a representation of total GH production we measured GH levels in 24-hour urine samples. We found an average of 8.6 ± 1.2 ng/day in our subjects, which fits within the 1–81 ng/day reported in healthy populations [78]. The consensus in the literature is that basal GH levels do not change following exercise training in healthy populations [69, 79, 80]. Therefore, our lack of change fits with the current literature.

Our resting IGF-1 levels were in the lower range of normal values for uninfected populations. Our results show no change in resting levels of IGF-1 following the exercise intervention. This finding, in combination with the reported presence of a change in elderly men [81] that often suffer the same decrease in lean tissue mass as HIV-infected persons, makes the absence of a change in resting IGF-1 levels in our population somewhat surprising. However, other clinical populations have failed to show a change in IGF-1 levels following exercise training, and some have even reported a decrease [82]. 

In addition to the lack of a change in IGF-1, the major binding protein, IGFBP-3, was also unchanged following our 6-week moderate-intensity exercise training intervention. The effects of individual exercise sessions on circulating IGFBP-3 levels are just now being realized, and the effect of exercise training on resting IGFBP-3 is largely unknown. Individual exercise sessions of moderate-intensity exercise appear to increase IGFBP-3 levels [83]. Combined with the increased IGFBP-3 proteolysis reported in HIV-infected persons, we anticipated an increase in resting IGFBP-3 levels. However, we did not see a change in IGFBP-3 following our intervention, and we found IGFBP-3 levels to be normal.

It appears that training status is a factor in the response of resting cortisol levels to exercise training. Prolonged exercise training does not appear to influence cortisol levels in trained persons [84, 85]; however, cortisol was lower following training in many populations, including sedentary adult males. Thus, it was unexpected that we did not see decreases in 24-hour cortisol levels following exercise training in our exercise naïve population. Additionally, it should be noted that resting cortisol levels in our population were within a normal range. However, our untrained population did exhibit a decrease in salivary cortisol following training at the wake time point and showed a trend for a decrease 1 hour after waking. 

 The literature clearly demonstrates that persons infected with HIV are prone to multiple hormone and cytokine abnormalities. However, the use of ART, which slows disease progression, can also normalize some of the hormone and cytokine levels in this population [86]. Thus, in our population receiving ART, and in whom a majority classified themselves as HIV-positive and asymptomatic, it is logical to see mostly normal hormone and cytokine profiles. Therefore, it is possible that these normal circulating levels explain the lack of changes following the exercise intervention. 

A limitation that affects many training studies, not just those involving HIV-infected persons, is subject retention and adherence to exercise. The high incidence of opportunistic infections in this population adds difficulty to subject retention, as does the poverty and lack of reliable transportation that so often is present in this population. Further, the stigma of HIV that is pervasive in this country dissuades many HIV-infected persons from responding to research opportunities. Given these circumstances, plus the 3–5 weekly exercise sessions required in other studies, poor retention rates and small sample sizes can be explained. However, our 77% retention rate suggests that (1) our sampled population was not burdened with the problems of other studied HIV-infected populations, (2) our twice weekly sessions were manageable, and (3) our moderate-intensity exercise regimen lessened the fatigue and muscle soreness that accompanies higher intensity regimens.

## 5. Conclusion

This research further strengthens the evidence for the safety and efficacy of prescribing resistance and aerobic exercise to HIV-infected men. No adverse events were reported, and there was a very low attrition rate. The only circulating hormone or cytokine we found in abnormal levels was IL-1-*β*, which was more than twice as high as the upper limit of the normal range. Aside from IL-1-*β*, our findings of “normal” circulating levels of hormones and cytokines in our population may explain our inability to detect changes in all these variables, except cortisol. It may be that those HIV-infected persons experiencing chronic low-grade inflammation may respond differently to the prescribed exercise intervention. Even in healthy populations, it is common to not detect changes in these variables, thus our HIV-infected population may be responding to exercise training the same as uninfected persons. Also, these data suggest that the benefits of exercise training in HIV-infected men using ART may be attributable to the transient decrease in resting CORT, the accumulation of hormone and cytokine changes following individual exercise sessions, or to factors yet to be investigated. Additionally, these results indicate that low-volume moderate-intensity exercise training, consistent with ACSM guidelines, is sufficient to produce beneficial physiological adaptations and the resulting performance improvements (increase in upper and lower body strength) in HIV-infected men receiving ART. Further, this study is the first to report differential effects of exercise training based on preintervention body composition. Both the +20 and −20 groups realized the health benefits of increasing lean tissue mass, without gaining fat mass, while the +20 group also experienced the health benefit of losing trunk fat. Taken together, these results provide evidence for the effectiveness of combined moderate-intensity aerobic and resistance exercise training at decreasing the risk for cardiometabolic diseases and increasing lean tissue mass; thus, strongly suggesting its inclusion in the standardized treatment plan of those infected with HIV-1.

## Figures and Tables

**Table 1:** Demographic data for subjects who completed the training study.

Exercise (MOD) group (*N* = 16)	Variable	Control (CON) group (*N* = 10)
43.1 ± 1.3	Age	46.6 ± 1.5

12	African American	6
4	Caucasian	4

373 ± 60 (7)	T-cell count	406 ± 76 (8)

8	HIV + symptomatic	6
3	HIV − symptomatic	1
3	AIDS	2
2	Did not answer	1

2	Excellent health	0
9	Good health	7
2	Fair health	2
1	Poor health	0
2	Did not answer	1

HIV status is based on 1993 CDC criteria.

**Table 2:** Demographic data for subjects who completed body composition assessment.

Exercise group (*N* = 31)	Variable	control Group (*N* = 27)
43.6 ± 1.3	Age	45.1 ± 1.5

20	African American	17
10	Caucasian	10
1	Other	0

28	ART Yes	25
3	ART No	2

17	HIV + symptomatic	19
4	HIV − symptomatic	1
7	AIDS	4
3	Did not answer	3

6	Excellent health	2
18	Good health	18
4	Fair hearth	6
1	Poor health	0
2	Did not answer	1

HIV status is based on 1993 CDC criteria. “ART Yes” refers to those subjects who reported taking at least 1 antiretroviral therapy during the study period.

**Table 3:** Resting hormone values and normal ranges.

Variable	MOD group (*N* = 16)			CON group (*N* = 10)			Normal range
	Pre	Post	*P* value	Pre	Post	*P* value	
IL-6 (pg/mL)	3.6 ± 1.0	3.1 ± 0.6	.6	3.6 ± 1.0	6.7 ± 2.0	.08	1–10
IL-1-*β* (pg/mL)	5.9 ± 0.5	7.5 ± 1.7	.9	6.2 ± 0.6	6.1 ± 0.5	.3	0–2.5
sTNFrII (ng/mL)	2.2 ± 0.3	2.4 ± 0.4	.6	2.6 ± 0.5	2.5 ± 0.5	.4	0.28–7
IGF-1 (ng/mL)	128 ± 10.5	132 ± 10.8	.4	118 ± 10.7	137 ± 17.5	.2	100–500
IGFBP-3 (ng/mL)	1897 ± 224	1934 ± 871	.7	1824 ± 247	1769 ± 218	.6	835–3778
Growth hormone (ng/day)	8.9 ± 1.7	7.1 ± 1.2	.3	15.1 ± 7.1	18.9 ± 11.8	.5	1–81
Cortisol (*μ*g/mL)	55.6 ± 5.9	61.2±9	.3	45.5 ± 7.2	57.7 ± 8.8	.1	10–100
Testosterone (saliva) (pg/mL)	101.1 ± 16.4	65.1 ± 4.7	.06	95.1 ± 29.3	61.6 ± 8.9	.3	30–100

**Table 4:** Body composition of exercise and control group subjects.

	Exercise group (*N* = 31)			Control group (*N* = 27)		
	Pre	Post	*P* value	Pre	Post	*P* value
Height (m)	1.76 ± 0.01	1.76 ± 0.01	—	1.74 ± 0.02	1.74 ± 0.02	—
Mass (kg)	71.4 ± 2.1	71.8 ± 2.1	.08	72.5 ± 2.5	72.9 ± 2.5	.847
BMI (m/kg)	23.1	23.2		24.0	24.1	
LTM (kg)	56.3 ± 1.1	57.1 ± 1.1	<.001*	55.9 ± 1.15	55.9 ± 1.6	.915
FM (kg)	14.3 ± 1.9	14.1 ± 1.9	.295	14.1 ± 1.7	13.9 ± 1.7	.178
% Total body fat	18.5 ± 1.9	18.2 ± 1.8	.182	18.1 ± 1.8	18.0 ± 1.8	.317
% Fat trunk	21.7 ± 2.2	21.1 ± 2.1	.058	21.7 ± 2.1	21.7 ± 2.1	.239
% Fat ARM	13.7 ± 1.8	13.7 ± 1.8	1.0	14.1 ± 1.5	14.1 ± 1.7	.683
% FatLEG	16.3 ± 1.9	15.9 ± 1.9	.347	16.6 ± 1.7	16.0 ± 1.7	.847
LTM ARM (kg)	6.8 ± 0.2	6.9 ± 0.2	.244	6.8 ± 0.2	6.9 ± 0.2	1.0
LTM LEG (kg)	18.4 ± 0.5	18.8 ± 0.5	.107	18.3 ± 0.4	18.4 ± 0.4	.811
LTM TRK (kg)	27.2 ± 0.7	27.4 ± 0.6	.430	27.3 ± 0.9	27.1 ± 0.7	.695

*Indicates a significant change within groups from pre- to postintervention.

**Table 5:** Body composition of exercise subjects above and below 20% body fat. Body composition variables, and within group changes, of exercise subjects as they are stratified into groups of above 20% total body fat (+20) and below 20% total body fat (−20), based on preintervention DXA testing.

	+20% Fat group (*N* = 14)			−20% Fat group (*N* = 17)		
	Pre	Post	*P* value	Pre	Post	*P* value
Height (m)	1.76 ± 0.01	1.76 ± 0.01	—	1.74 ± 0.02	1.74 ± 0.02	—
Mass (kg)	78.1 ± 2.7	78.0 ± 3.0	.665	65.8 ± 2.4	66.7 ± 2.4	.007*
BMI (m/kg)	25.4 ± 0.9	25.3 ± 1.0	.664	21.3 ± 0.8	21.6 ± 0.9	.007*
LTM (kg)	56.4 ± 1.7	57.1 ± 1.7	.027*	56.3 ± 1.5	57.1 ± 1.5	.014*
FM (kg)	23.4 ± 2.4	22.71 ± 2.4	.02*	6.7 ± 0.9	7.0 ± 1.0	.143
% Total Body Fat	28.6 ± 1.5	27.7 ± 1.6	.009*	10.1 ± 1.1	10.4 ± 1.1	.269
% Fat Trunk	33.7 ± 1.3	32.2 ± 1.4	.001*	11.8 ± 1.5	12.1 ± 1.5	.299
% Fat ARM	22.3 ± 2.4	22.0 ± 2.4	.619	6.6 ± 0.8	6.8 ± 1.1	.757
% FatLEG	24.3 ± 2.7	24.0 ± 2.8	.269	9.7 ± 1.2	9.3 ± 0.9	.578
LTM ARM (kg)	6.9 ± 0.3	6.9 ± 0.3	.746	6.8 ± 0.3	6.9 ± 0.3	.189
LTM LEG (kg)	18.4±.0.6	18.6 ± 0.6	.660	18.5 ± 0.7	19.1 ± 0.8	.014*
LTM TRK (kg)	27.3 ± 1.1	27.8 ± 0.9	.268	27.0 ± 0.8	27.0 ± 0.7	.898

*Indicates a significant change from pre- to postintervention within groups.

**Table 6:** Strength changes in exercise and control group subjects. Peak strength results, and within group changes, for the exercise and control groups.

Exercise	Exercise Group (*n* = 17)			Control Group (*n* = 11)		
	Pre	Post	*P* Value	Pre	Post	*P* Value
Leg Extension	153.8 ± 13.3	185.3 ± 13.5	<.001*	140 ± 11.3	161.4 ± 13.9	.03*
Leg Curl	120.4 ± 9	143.2 ± 7.9	.001*	105.5 ± 6.1	128.2 ± 7.1	.006*
Lat Pull	132.4 ± 7.4	148.8 ± 7.2	<.001*	120.9 ± 7.6	124.1 ± 7.4	.532
Chest Press	118.5 ± 6.2	135.3 ± 7	<.001*	111.6 ± 8.7	109.6 ± 6.1	.782

*Indicates a significant change from pre to post test.
